# The Concept of Psychologically Informed Heart Transplantation Care

**DOI:** 10.3390/jcm15041592

**Published:** 2026-02-18

**Authors:** Alexandra Assabiny, Zsófia Ocsovszky, Blanka Ehrenberger, József Otohal, Orsolya Papp-Zipernovszky, Béla Merkely, Balázs Sax, György Purebl

**Affiliations:** 1 Heart and Vascular Center, Semmelweis University, 1085 Budapest, Hungary; ocsovszky.zsofia@semmelweis.hu (Z.O.);; 2 Institute of Behavioural Sciences, Semmelweis University, 1085 Budapest, Hungary

**Keywords:** psychologically informed care, psychosocial factors, heart transplantation, multidisciplinary care

## Abstract

The success of heart transplantation (HTX) is profoundly influenced by various psychosocial factors, underscoring the necessity of a multidisciplinary approach throughout the patient’s journey. Relevant psychosocial factors, such as adherence to complex post-transplant regimens, psychological status (e.g., risk of anxiety, depression), and the long-term goal of social and professional reintegration (return to work), significantly impact post-transplant outcomes. Current guidelines strongly recommend a multidisciplinary team, including psychologists, social workers, and other specialized non-medical professionals, as their involvement is associated with better quality of care and improved long-term outcomes. This paper synthesizes the evidence underlying psychologically informed heart transplantation care, highlighting the integration of fundamental psychological principles into routine medical practice. This approach features a genuine transdisciplinary collaboration, extending beyond traditional medical care. The implementation framework includes adopting the World Health Organization’s Multidimensional Adherence Model, tailored to the HTX population, and a spectrum approach to mental health, facilitating unified assessment and care aligned with the competencies of different team members. Furthermore, the collaborative team is trained in Low-Intensity Psychological Interventions, which are simple and effective; these can be embedded into routine clinical pathways by various healthcare staff to quickly address anxiety, adherence, and suboptimal health behaviors. By applying the concept of psychologically informed care, HTX centers can facilitate the genuine integration of interdisciplinary collaboration, ensuring comprehensive physical and psychosocial support for recipients.

## 1. Relevant Psychosocial Factors and Aspects of the Heart Transplantation Journey

Numerous psychosocial factors influence the success of heart transplantation. Therefore, as part of the routine pre-transplant assessment, a complex psychosocial evaluation is recommended before a patient’s activation on the waiting list [1]. Based on the International Society for Heart and Lung Transplantation’s guideline [1] and “The 2018 ISHLT/APM/AST/ICCAC/STSW Recommendations for the Psychosocial Evaluation of Adult Cardiothoracic Transplant Candidates and Candidates for Long-term Mechanical Circulatory Support” [2] consensus document, the evaluation should target three main topics:

1. Predictors of poor post-transplant outcomes;
2. Factors influencing patient knowledge, understanding, and engagement in the informed decision-making process;
3. Personal, social, and environmental resources and possibilities.

The consensus statement deeply discusses these topics: predictors of post-transplant outcome include adherence, psychological and familial history, as well as currently existing psychiatric diseases (depression, anxiety, personality disorders), and current and past substance abuse. Cognitive condition, capacity to understand information, knowledge of the disease, and treatment options should be assessed as factors influencing decision making. Evaluation should also reveal the patient’s emotions regarding the current situation, coping strategies, level of education, and relationship status. Availability and stability of social support are essential resources throughout the transplant journey [2]. The importance of these factors is underscored by empirical evidence [3,4,5,6,7,8]. Many of them have been shown to influence transplant outcome, risk of graft loss, adherence, and mortality. Moreover, most of these psychosocial factors are interlinked, making the evaluation a complex screening process, which must be tailored to patients’ medical status and ability to participate [2]. Therefore, the authors of the consensus document highlight that universal, strict guidelines are inappropriate in this field. It can be used as flexible guidance that supports comprehensive evaluations across diverse patients and transplant program settings [2]. The consensus document recommends that the evaluator of psychosocial status must possess the requisite competence—derived from appropriate qualifications, knowledge, and clinical experience—to conduct and communicate a sensitive and accurate assessment. No single professional discipline or training pathway is inherently superior for fulfilling this role. However, the evaluator should have formal training in a healthcare profession that is directly relevant to the psychosocial evaluation’s scope and content. Continued participation in educational and professional development activities should be supported to ensure ongoing skill enhancement and to maintain compliance with professional standards. To promote consistency and completeness, the expert consensus recommends the use of a standardized template or checklist encompassing all key elements of the psychosocial evaluation, thereby facilitating systematic coverage, structured documentation, and comprehensive summary reporting. Such tools may be developed locally or alternatively, and may be adapted from existing published instruments designed for transplant candidate assessment, such as The Stanford Integrated Psychosocial Assessment for Transplantation (SIPAT) [9], the Psychosocial Assessment of Candidates for Transplantation (PACT) [10] and the Transplant Evaluation Rating Scale (TERS) [11]. In clinical practice, SIPAT results are associated with non-adherence [12] and high-risk scores predict reduced long-term post-transplant survival [13]. Summarizing the guidelines, we would like to point out some discrepancies regarding the lack of psychometric aspects of the applied assessment tools. This raises the question of generalizability [14].

Maintaining adherence after transplantation is crucial. Patients must comply with complex follow-up care such as hygiene, dietary restrictions, and an immunosuppressive drug regimen, which all significantly affect their daily lives [1,2]. Psychological status is also an important component of adaptation following transplantation. Coping with difficult feelings and distress during adaptation to a new life setting and re-adaptation to the social world, as well as returning to the life left behind, can be challenging [15]. Finding meaning and creating a new narrative of life, integrating the history of illness, is just part of the work that has to be done. Heart transplant recipients have a higher risk of developing new mood and anxiety disorders in the first year. Longitudinal studies have shown that distress caused changes in body image [16], magical thinking about inheriting the donors’ personality [17,18,19], fear of death [20], financial problems, and family difficulties are frequent problems among patients. Keeping a constant focus on the risk of allograft rejection and immunosuppressive medication regimen may lead to anxiety and depression. Prevalence of post-traumatic stress disorder is also elevated [21,22]. Qualitative reports from organ transplant recipients suggest that psychological distress may partly stem from (mis)conceptions about the donor and/or the transplanted organ [23]. Despite its importance, this is still an understudied phenomenon with limited systematic evidence and no established assessment tools [23]. Previously described as “fantasies about the donor” or “fantasies about the organ”, current literature cites this as donor and donation images (DDI) [23]. The latest large cross-sectional study [24] found a very high prevalence of DDI (91%) resulting from overall higher emotional activation of the patient before the transplantation. The authors consider DDI as part of coping with the transplantation process.

The final goal of post-transplant care is complex rehabilitation and social reintegration. In the 2009 collection of studies edited by Könczei [25], the author references that DeLisa and colleagues describes rehabilitation, asthe process that assists an individual in achieving their full potential in physical, psychological, social, professional, extracurricular, and educational aspects in a manner consistent with the nature of their somatic impairments, the limitations imposed by their environment, their own desires, and their visions of life. When placing the above in the context of heart transplant care, it is necessary to provide both physical assistance and psychosocial support to recipients during rehabilitation and their reintegration into their families, immediate environments, and society. Rivera et al. [26] found in their international literature review that while 70–86.6% of patients were employed before heart transplantation, only 30–60% returned to work after the surgery. In addition to physical condition and capacity, psychosocial factors were identified as predictors of employability. The current care guidelines [27] state that returning to work is a rehabilitation goal, as employment is associated with better post-transplant quality of life. Thus, it is recommended to facilitate an early return to work, considering physical condition, even if it is part time.

A crucial part of a comprehensive approach to rehabilitation is providing systemic psychosocial support throughout the care process. In the long term, collaboration between the transplant care system and a palliative team is essential to meet patients’ physical, emotional, and spiritual needs [28].

For optimal rehabilitation and long-term outcomes, medication adherence, lifestyle compliance, and psychological well-being must be effectively supported and optimized. According to the current guidelines [27], post-transplant care remains a task for a multidisciplinary team, which conducts regular meetings involving all disciplines. Integrating social workers, psychologists, psychiatrists, pharmacists and dieticians into the teamwork is recommended. A new recommendation highlights the role of a nurse responsible for coordinating care and integrating physical therapists and occupational therapists into post-transplant care for early mobilization and rehabilitation. The involvement of specialized, non-medical professionals in care could lead to improved long-term transplant outcomes, while further studies are needed in the future to evaluate the effectiveness of interventions that affect adherence [27].

The practical implementation of multidisciplinary teamwork is influenced by numerous factors and can vary widely. According to international literature, the framework design and operational model have a significant impact on the quality of care. This is supported by findings from Cajita et al. [29], who examined the practical implementation of multidisciplinary teamwork as recommended by guidelines and their association with patient-perceived quality of care. In their study, a secondary analysis was conducted using data from the BRIGHT (Building Research Initiative Group: Chronic Illness Management and Adherence in Transplantation) international study, which included datasets from 36 heart transplantation centers. The analysis incorporated data from 1397 recipients and 36 center directors. For the purpose of the study, multidisciplinary care was defined as a team consisting of physicians, nurses, and at least one additional healthcare professional (such as a social worker, psychiatrist, psychologist, pharmacist, dietician, physical therapist, or occupational therapist) who routinely participated in outpatient follow-up care. Among the examined centers, 80.6% had a multidisciplinary team. Routine outpatient care integration varied, with 47% of providers incorporating social workers, 44% pharmacists, 31% psychologists, 28% physical therapists, 22% dietitians, 5.6% psychiatrists, and 5.6% occupational therapists. A key finding of the study was that care provided by a multidisciplinary team was associated with a higher level of perceived disease management among recipients. Roussel et al. [30], in the context of transplantation, demonstrated that optimizing their multidisciplinary care practices led to a significant improvement in post-transplant hospitalization duration (median reduction of 37%) and 30-day rehospitalization rates (decrease of 33%).

## 2. Psychologically Informed Care

The concept of psychologically informed patient care refers to the fundamental integration of basic psychological principles into medical care [31]. It incorporates a genuine transdisciplinary approach through the interaction of psychology and medicine, resulting in enhanced patient care beyond the borders of traditional medical care. The psychologically informed care concept has been applied mainly in the management of chronic pain [32,33] and musculoskeletal disorders [34,35].

According to Dekker et al. [31], integrating psychological principles into daily patient care can be achieved directly or indirectly. In the direct way, psychologists deliver evidence-based psychological interventions, whereas in the indirect way they support healthcare professionals in applying psychological principles to their daily work, thereby enabling the provision of psychologically informed patient care. Targeted training is provided to healthcare professionals, enhancing specific abilities and skills (e.g., active listening, empathy, communication, stress and anxiety reduction), and may improve the effectiveness of clinical interventions in the form of indirect psychological care.

Relevant literature highlights shorter and long-term positive outcomes resulting from integrated psychological support. Mumford et al. [36] highlighted in 1982, after reviewing 34 studies, that patients who received psychological interventions provided by healthcare workers, psychiatrists, and psychologists adapted better to the medical situation compared to the control group, which received usual care. According to Holdgaard et al. [37], brief cognitive-behavioral therapy provided by cardiac nurses to patients with coronary artery disease has been shown to reduce anxiety and depression scores, improve Health-Related Quality of Life and adherence to cardiac rehabilitation, as well as decrease cardiology-related readmissions. Jiang et al. [38] conducted a meta-analysis on the effects of psychological interventions on self-care, depression, anxiety levels and health-related quality of life, including the physical function of patients with chronic heart failure. The authors identified five types of interventions, including cognitive-behavioral therapy, motivational interviewing, multi-component interventions (disease education, strategies to elicit social support, and self-monitoring of symptoms and behavior), stress management, and supportive counseling. In the included studies, the interventions were delivered mainly by nurses, psychologists, researchers, and other allied health professionals. According to their findings, psychological interventions tend to improve self-care in chronic heart failure patients without clinical depression and cognitive impairment, as well as short-term health-related quality of life. They highlight the importance of nurses in patient education and secondary prevention, as they have more opportunities for patient contact [38]. Tyrer et al. demonstrate in their research that trained nurses can be effective therapists in medical settings for treating and reducing anxiety [39]. These trainings aim to enhance empathy, develop the ability to recognize the patient’s emotions and emotional distress, and empower healthcare providers with specific communication techniques and minimally invasive psychological interventions [40,41].

## 3. An Example of Practical Implementation of Psychologically Informed Transplant Care

Basically, the Semmelweis University Heart and Vascular Center’s heart failure and heart transplant multidisciplinary team comprises experts from various disciplines, including cardiologists, cardiac surgeons, anesthesiologists, intensive care specialists, coordinators, nurses, psychologists, psychiatrists, physiotherapists, dietitians, and clinical pharmacists. However, the integration of psychosocial aspects into the daily medical practice needed further effort during the last seven years: to ensure the most effective collaborative teamwork within our center, we have established a practical framework based on existing literature and methodologies. This serves as a common language in everyday patient management and as training material. In the next paragraphs, we introduce its basic principles and relevant related literature. Our description is intended to serve as an illustrative model of psychologically informed care implementation, rather than a formally validated approach.

### 3.1. Multidimensional Adherence Model Adapted to Heart Transplant Care

As previously discussed, adherence is a crucial element of the heart transplantation journey, which must be evaluated throughout its entire duration. At the same time, adherence is a complex construct influenced by various factors.

The Multidimensional Adherence Model (MAM) of the World Health Organization (WHO) identifies patient-related, condition-related, therapy-related, healthcare team or system-related, and socioeconomic factors [42]. We adapted the Multidimensional Adherence Model for the heart transplantation population (Table 1).

Based on this model, adherence is a multifaceted construct with interacting components. Accordingly, adherence interventions must target a broad range of health domains. In a traditional setting, psychological interventions provided by psychologists or psychiatrists can focus on individual factors of adherence (e.g., depression) and address these mental conditions as comorbidities of somatic disease. Psychologically informed care reflects the interplay among somatic, mental and social status, and in this way can have a beneficial impact on a broader range of health domains [31], which is crucial during heart transplant care. Based on these, we review below the implementation steps undertaken at our center for developing the psychologically informed knowledge and skills of the medical team to strengthen the indirect ways of care.

### 3.2. The Spectrum Approach

**Figure 1:**
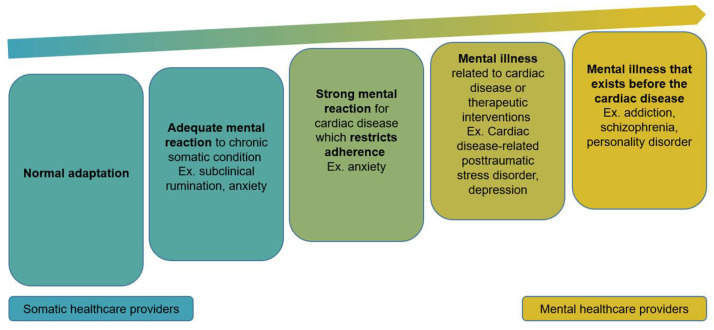
The spectrum approach. Cardiac disease and post-transplant somatic conditions can elicit a range of psychological reactions, from normal adaptation [67,68,69] and adequate mental responses [70] to strong reactions that restrict adherence [71,72,73], disease- or treatment-related mental illness [21,22,74,75,76,77,78,79], and pre-existing mental disorders [80,81,82,83,84,85,86]. The intensity of the response alone does not define pathology; key differentiators include duration, proportionality, functional impact on treatment adherence, and daily functioning. Somatic healthcare providers typically manage lower-level reactions, while mental healthcare providers are engaged as the severity or complexity of mental health conditions increases [21,22,67,68,69,70,71,72,73,74,75,76,77,78,79,80,81,82,83,84,85,86].

To facilitate the identification of psychological status and the common nomenclature in a multidisciplinary, psychologically informed care setting, we conceptualized heart failure and heart transplant patients’ mental health and healthcare providers’ competence as a spectrum phenomenon (Figure 1). The spectrum-based approach supports medical professionals from various disciplines in identifying the patient’s psychological condition, conducting a unified, nuanced, and clinically relevant assessment, and providing care that aligns with their respective competencies.

This spectrum approach highlights the core competencies of somatic medical staff (e.g., physicians, nurses, coordinators, etc.): at every point on the spectrum, our task and responsibility is to recognize and empathize. In addition, using a psychologically informed care conception, we defined psychological competencies of the medical team members. A promising approach to increase efficacy and to improve cost effectiveness in the management of cardiac patients to implement low-intensity psychological interventions (LIPIs). Originally developed for the United Kingdom National Health Service by the “Increase Access to Psychological Therapies” program [87,88] and also for the implementation of accessible psychological support in low- and middle-income countries by the WHO [89,90], LIPIs can provide simple but effective psychological interventions that can be easily delivered by any hospital staff beyond psychologists. These interventions can serve as a rapid response to various transdiagnostic psychological problems, non-adherence or suboptimal health behaviors. It is essential to note that most LIPIs have been developed from cognitive-behavioral therapy, a psychosocial approach that has been demonstrated to be effective in numerous studies involving patients with cardiac conditions [91,92]. To ensure the highest quality of care, we involved the transplant cardiology team into a consciously designed LIPI training system. Beyond that, one of the cardiologists completed training as a mental health counselor and addiction counselor.

The spectrum approach involves those mental health conditions, diseases and illnesses that impact somatic care. For effective somatic treatment, the involvement of psychologists or psychiatrists can be beneficial [93]. Accordingly, psychologists were integrated into the medical team. These professionals have backgrounds in relevant disciplines, including clinical psychology, health psychology, addiction counseling, and psychotherapy. We have established structured referral criteria that support step-care decision making and ensure the appropriate matching of patient needs to the level of psychological or psychiatric intervention.

## 4. Barriers of Practical Implementation

During the implementation of new and innovative processes, e.g., psychologically informed care, there can be several theoretical barriers. Based on implementation science theoretical frameworks, these hindering factors can occur at different levels of the healthcare system [94,95], for example, (a) organizational barriers, including limited financial resources, reimbursement arrangements, competing clinical priorities, and insufficient institutional support; (b) the lack of standardized training programs and protected time for staff education, restricting clinicians from acquiring and maintaining the necessary competencies; and (c) workforce constraints, such as staffing shortages, high workload, and lack of individual motivation. All these can limit the opportunities for an interdisciplinary collaboration and the consistent application of psychologically informed principles in routine clinical practice [95].

## 5. Conclusions

It can be concluded that, beyond somatic interventions, significant emphasis must be placed on psychosocial factors through the delivery of care during the heart transplantation process. In heart transplantation care, integrating psychosocial considerations is indispensable. Management within a multidisciplinary team provides an appropriate framework for accomplishing these complex tasks; however, for successful integration into everyday clinical practice, the presence of domain experts alone is insufficient. Establishing a shared language and a unified conceptual approach is also essential. Applying the concept of psychologically informed care within the transplantation field may facilitate the genuine implementation of interdisciplinary collaboration in daily clinical practice, thereby supporting the care of individuals involved in the heart transplantation process.

## Figures and Tables

**Table 1:** Relevant psychosocial factors during the heart transplant journey applied to the WHO Multidimensional Adherence Model (MAM) in a multidisciplinary care setting.

MAM Dimension	Factor- Related Content	HTX Specificity	Potential Barriers to Adherence	Evidence-Based Interventions
Patient-related	Gender, Age, Attitudes, Knowledge [42]	misbeliefs of the donated heart: donor and donation images; psychological acceptance of the new heart; emotional connection with the donor (feeling of guilt); recovered patient phenomena; misbeliefs and misinformation about medications; HTX-specific patient education; cognitive status; psychological conditions, psychiatric comorbidities [4,43,44,45,46,47,48,49,50]	Lack of motivation or self-efficacy; beliefs and concerns; cognitive impairments; depression, anxiety [4,43,44,45,46,47,48,49,50]	Motivational interviewing, problem-solving training, digital reminders, combined education and behavior change counseling, psychological support [4,43,44,45,46,47,48,49,50]
Condition-related	Symptom severity, Comorbidity [42]	progression of rehabilitation–increase in physical capacity; body and visceral sensations connected to the new heart; affective status connected to somatic experiences; post-transplant affective and anxiety disorders, recognizing the signs of rejection or other malformations that can cause rejection; [50,51,52,53,54,55]	Asymptomatic chronic conditions → low risk perception; comorbidities; disease fluctuation, post-transplant depression, and its effect on cognitive function [50,51,52,53,54,55]	Self-monitoring (e.g., home blood pressure/glucose); regular feedback; condition-specific education; psychological check-ups, parallel to medical check-ups; and supportive therapy [50,51,52,53,54,55]
Treatment-related	Complexity of Medication, Dose, Frequency [42]	life-long immunosuppression; deep understanding of medications; HTX-specific diet and rules; medication flavor and dosage; type of formulations, frequency of administration, product-related considerations; multimorbidity caused by medication side effects [56,57,58,59]	Polypharmacy; complex dosing regimen; side effects; parenteral/compound formulations [56,57,58,59]	Regimen simplification (once daily; fixed-dose combinations); packaged dosing; reminders; side effect management; pharmacist review, clinical pharmacist in HTX care team [56,57,58,59]
Healthcare team/system- related	Patient–provider relationship [42]	lack of HTX-specific training; lack of HTX-specific research evidence; lack of HTX-specific training material and protocols; poor physician–patient relationship, lack of continuity in medical practice and availability of the medical team; lack of time for healthcare providers to inform patients [60,61,62]	Fragmented care, access difficulties, poor doctor–patient communication, non-standardized processes [60,61,62]	Team-based care, pharmaceutical care, standardized protocols, simplified appointment scheduling, and shared decision making [60,61,62]
Social/economic factors	Education, Ethnicity, Financial status, Social support [42]	high ratio of heart muscle damage leading to HTX is linked to socioeconomic status; high ratio of HTX in vulnerable groups; cultural aspects of illness; poor living conditions; low level of health literacy; culture of empathy and providing support; misbeliefs on HTX overprotection as a barrier to reintegration [8,30,63,64,65,66]	Low income, low health literacy, weak social support, and logistical or cost barriers [8,30,63,64,65,66]	Family/peer support programs, cost reduction (generic and subsidized drugs), a social worker on the team, health literacy intervention [8,30,63,64,65,66]

## Data Availability

The datasets generated and analyzed during the current study are not publicly available due to the presence of sensitive personal and health-related information and restrictions imposed by the institutional ethics committee and the General Data Protection Regulation (GDPR). Anonymized data may be available from the corresponding author on reasonable request and with permission of the relevant ethics committee.

## Abbreviations

The following abbreviations are used in this manuscript:

LIPI	Low-Intensity Psychological Interventions
MAM	Multidimensional Adherence Model
HTX	heart transplantation
WHO	World Health Organization
